# Stanniocalcin 2 (STC2): a universal tumour biomarker and a potential therapeutical target

**DOI:** 10.1186/s13046-022-02370-w

**Published:** 2022-05-02

**Authors:** Shuo Qie, Nianli Sang

**Affiliations:** 1 Department of Pathology, Tianjin Medical University Cancer Institute and Hospital, Tianjin, China; 2 National Clinical Research Center for Cancer, Tianjin, China; 3 Key Laboratory of Cancer Prevention and Therapy (Tianjin), Tianjin, China; 4 Tianjin’s Clinical Research Center for Cancer, Tianjin, China; 5 Department of Biology, Drexel University, PA Philadelphia, USA

**Keywords:** Stanniocalcin 2, Stress Response, Tumorigenesis, Tumour progression, Prognosis, Cancer therapy

## Abstract

Stanniocalcin 2 (STC2) is a glycoprotein which is expressed in a broad spectrum of tumour cells and tumour tissues derived from human breast, colorectum, stomach, esophagus, prostate, kidney, liver, bone, ovary, lung and so forth. The expression of STC2 is regulated at both transcriptional and post-transcriptional levels; particularly, STC2 is significantly stimulated under various stress conditions like ER stress, hypoxia and nutrient deprivation. Biologically, STC2 facilitates cells dealing with stress conditions and prevents apoptosis. Importantly, STC2 also promotes the development of acquired resistance to chemo- and radio- therapies. In addition, multiple groups have reported that STC2 overexpression promotes cell proliferation, migration and immune response. Therefore, the overexpression of STC2 is positively correlated with tumour growth, invasion, metastasis and patients’ prognosis, highlighting its potential as a biomarker and a therapeutic target. This review focuses on discussing the regulation, biological functions and clinical importance of STC2 in human cancers. Future perspectives in this field will also be discussed.

## Background

**Fig. 1:**
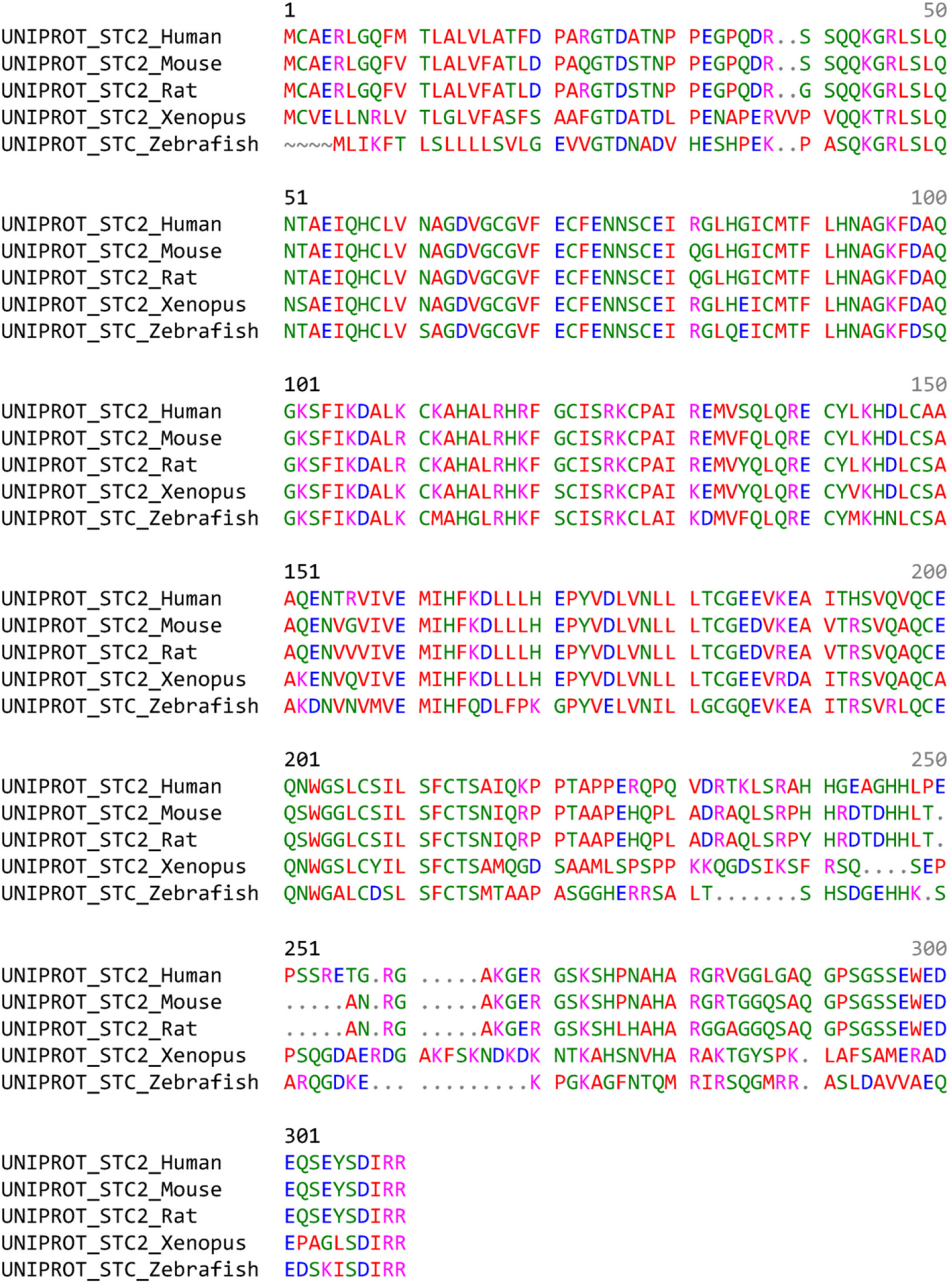
The comparaison of STC2 protein sequences among indicated species. It suggests highly phylogenetic consevation in different vertebrates

STC2 belongs to a highly conserved, secreted glycoprotein hormone family. The term “stanniocalcin” (STC) derives from the corpuscles of Stannius, the endocrine glands that are ventrally located on the surface of the fish kidney [1]. STC was initially reported to lower the intracellular calcium concentrations through compromising Ca^2+^ influx in fish [2–4]. Two isoforms of human STC have been identified: STC1 and STC2 [5, 6]. STC1, the human ortholog of fish STC, was identified through the differential display of genes related to cellular immortalization and the random sequencing of a fetal lung DNA library [7–11]. Human STC2, a paralog of STC1, was identified by searching the STC1 related Expressed Sequence Tag database [12–14]. STC2 shows 34% identity with human STC1 and eel STC based on sequencing analysis [14]. Phylogenetically, STC2 is highly conserved in vertebrates, including the most common animal models *Danio rerio, Xenopus tropicalis, Mus musculus, and Rattus norvegicus* (Fig. 1), suggesting a biologically important role of STC2 in species preservation during evolution and highlighting the animal models to study STC2.

Human STC1, a protein with 247 amino acids [15], is broadly expressed in a series of human tissues including kidney [16], ovary [17], bone [18], prostate [18], thyroid [18], neuron [19], muscle [20], etc. Biologically, STC1 is involved in physiological processes such as intestinal Ca^2+^ transport and organogenesis [9, 21–24], suggesting STC1 plays similar functions like its fish ortholog.

**Fig. 2:**
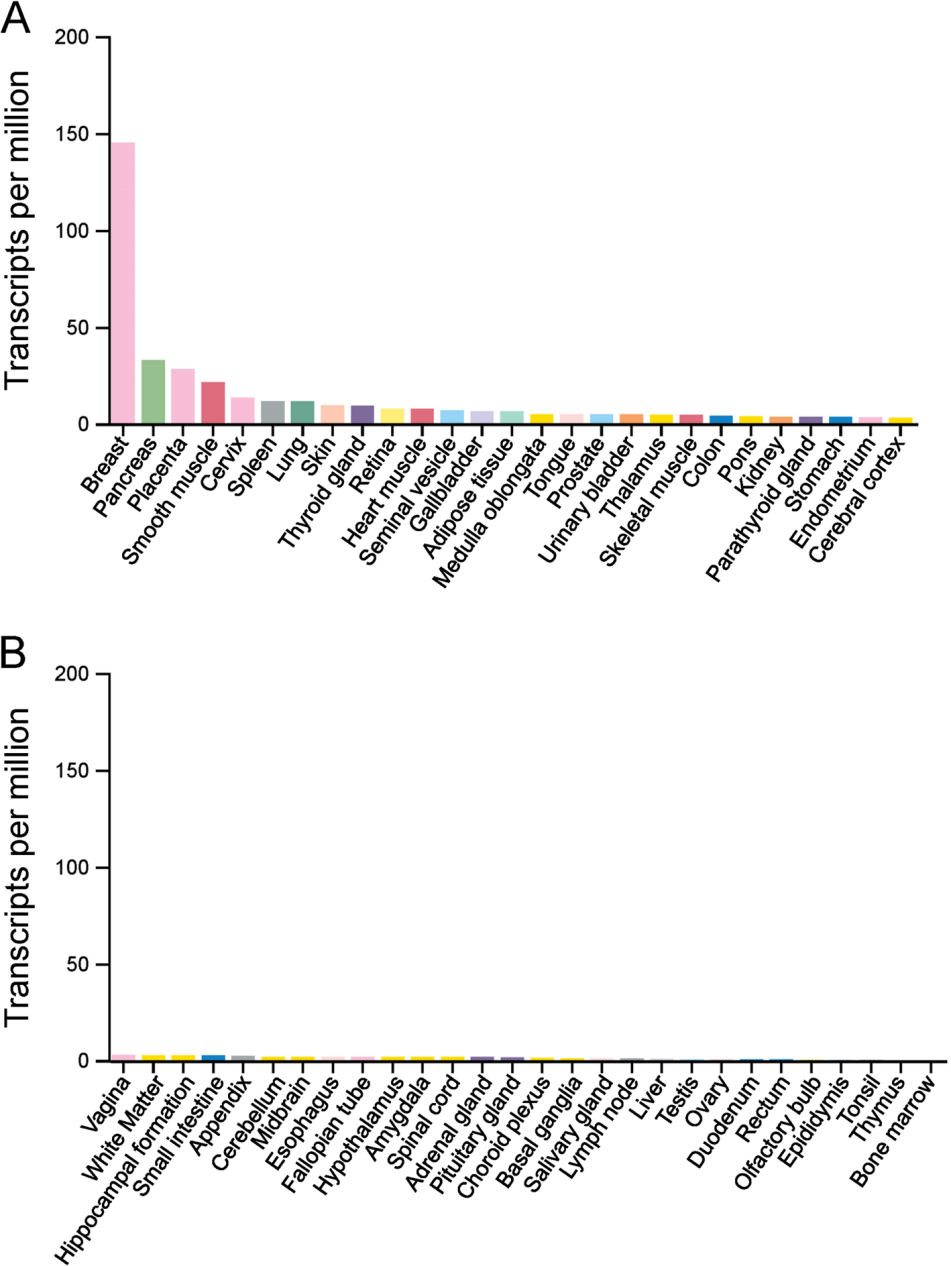
The mRNA expression of STC2 in human normal tissues. The panels A & B were generated using online tool: The Protein Atlas (https://www.proteinatlas.org/)

Human STC2 is a 302-amino acid (aa) protein, with aa1-24 as signal peptide for ER transportation. Considering its mRNA levels, STC2 is highly expressed in tissues of breast, muscle, heart, testis and pancreas under physiological conditions as indicated by The Human Protein Atlas [14], which is consistent with the RNA expression in normal human tissues (Fig. 2). Although STC1 has been reported to regulate multiple processes during tumour development and progression [15, 25–28], the different expression pattern of STC1 and STC2 indicates they may have different roles under physiological or pathological conditions.

Importantly, STC2 is broadly upregulated in human tumours including breast cancer [29–32], colorectal cancer [33], gastric cancer [34], esophageal squamous cell carcinoma (ESCC) [35], prostate cancer [36], renal cell carcinoma (RCC) [37], nasopharyngeal carcinoma [38], head and neck squamous cell carcinoma (HNSCC) [39], hepatocellular carcinoma (HCC) [40], osteosarcoma [41], ovarian cancer [42], lung cancer [43], pancreatic cancer [44], endometrial cancer [45], cervical cancer [46], neuroblastoma [47], gallbladder cancer [48], cholangiocarcinoma [49], and so forth. Clinicopathological investigations indicate the expression of STC2 is correlated with tumour development, tumour progression and patients’ prognosis (Table 1).

**Table 1:** The clinical importance of STC2 in human cancers

Tumours	Patient Numbers	Detection Methods	Clinical Importance
Breast cancer	110	qRT-PCR	Elevated STC2 mRNA levels are correlated with higher DFS rates [50]
	245	FISH	STC2 expression is associated with improved DFS rates [51]
	110	IHC	No relationship with progression free survival is demonstrated [52]
	985 + 979	Microarray	STC2 levels are correlated with good prognosis [53]
	699	IHC	No correlation is revealed between STC2 levels and tumour recurrence [54]
	477	IHC	STC2 is associated with favorable outcome in male breast cancer patients [55]
Colorectal cancer	139	qRT-PCR	High STC2 levels are correlated with poor prognosis [33]
	47	qRT-PCR	STC2 expression is associated with tumour size [56]
	77	IHC	STC2 levels are correlated with tumour stage and patients’ survival [57]
	383	Microarray	STC2 expression is associated with tumour stages [58]
	115	IHC	STC2 levels are correlated with tumour metastasis and disease stages [59]
	379	RNA-Seq	Elevated STC2 expression is associated with poor prognosis [60]
	202	qRT-PCR	High STC2 mRNA expression is correlated with poor postoperative survival [61]
Gastric cancer	108	qRT-PCR	STC2 mRNA levels are higher in cancer than those in normal gastric mucosa, and associated with tumour progression and poor prognosis [62]
	93	qRT-PCR	Blood STC2 levels are correlated with tumour progression and poor prognosis [63]
ESCC	70	qRT-PCR	Elevated STC2 mRNA levels are associated with lymph node metastasis and poor prognosis [35]
Prostate cancer	53	IHC	STC2 IHC staining score is correlated with high Gleason scores [36]
RCC	108	IHC	For all RCC, clear cell RCC, papillary RCC, patients with high cytosolic STC2 staining have a poor prognosis than those with low expression [37]
Nasopharyngeal carcinoma	94	IHC	Overall survival rate of patients with high STC2 levels is significantly lower than those with low STC2 expression [38]
	62	IHC	STC2 is a valuable prognostic marker for poor outcome [64]
	81	IHC	STC2 positive patients show poor outcome [65]
	111	RNA-Seq	STC2 is regarded as a biomarker for evaluating patients’ prognosis [66]
	68	IHC	STC2 expression is associated with poor progression [67]
	111	RNA-Seq	STC2 can be applied to predict the overall survival rates of patients [68]
HNSCC	298	IHC	Elevated STC2 expression is correlated with poor outcome [39]
	119	IHC	High STC2 expression is significantly associated with poor survival [69]
	498	RNA-Seq	High STC2 levels are correlated with poor prognosis [70]
HCC	240	IHC	STC2 expression is associated with poor survival [40]
	200	IHC	High STC2 is correlated with poor prognosis [71]
	258	IHC	High STC2 expression can be utilized to predict overall survival [72]
	364	RNA-Seq	STC2 expression is associated with poor prognosis of HCC [73]
Osteosarcoma	88	RNA-Seq	STC2 has an important influence on the survival of patients [41]
	88	RNA-Seq	STC2 is correlated with the patients’ prognosis and metastasis [74]
	87	RNA-Seq	Elevated STC2 levels are associated with poor prognosis [75]
Ovarian cancer	46	qRT-PCR	Overexpression of STC2 by vascular cells in ovarian cancer is correlated with decreased disease-free interval [42]
	278	IHC	STC2 is associated with tumour grade, tumour histotype and poor overall survival [76]
Lung cancer	115	IHC	STC2 mediates the resistance to EGFR tyrosine kinase inhibitors [43]
Pancreatic Cancer	98	qRT-PCR	STC2 expression is correlated with clinicopathological factors and patients’ prognosis [44]
Endometrial cancer	49	IHC	High STC2 expression is associated with poor relapse-free survival [45]
Cervical cancer	92	IHC	STC2 expression is correlated with shorter survival [46]
Neuroblastoma	50	qRT-PCR	STC2 mRNA levels are associated with higher clinical stages [47]
Gallbladder cancer	46 + 80	IHC	STC2 expression is correlated with clinical, pathological, and biological behaviors as well as poor prognosis of gallbladder cancer [48]
Cholangiocarcinoma	100	IHC	Positive expression of STC2 is associated with progression and poor clinical outcomes [49]

**Fig. 3:**
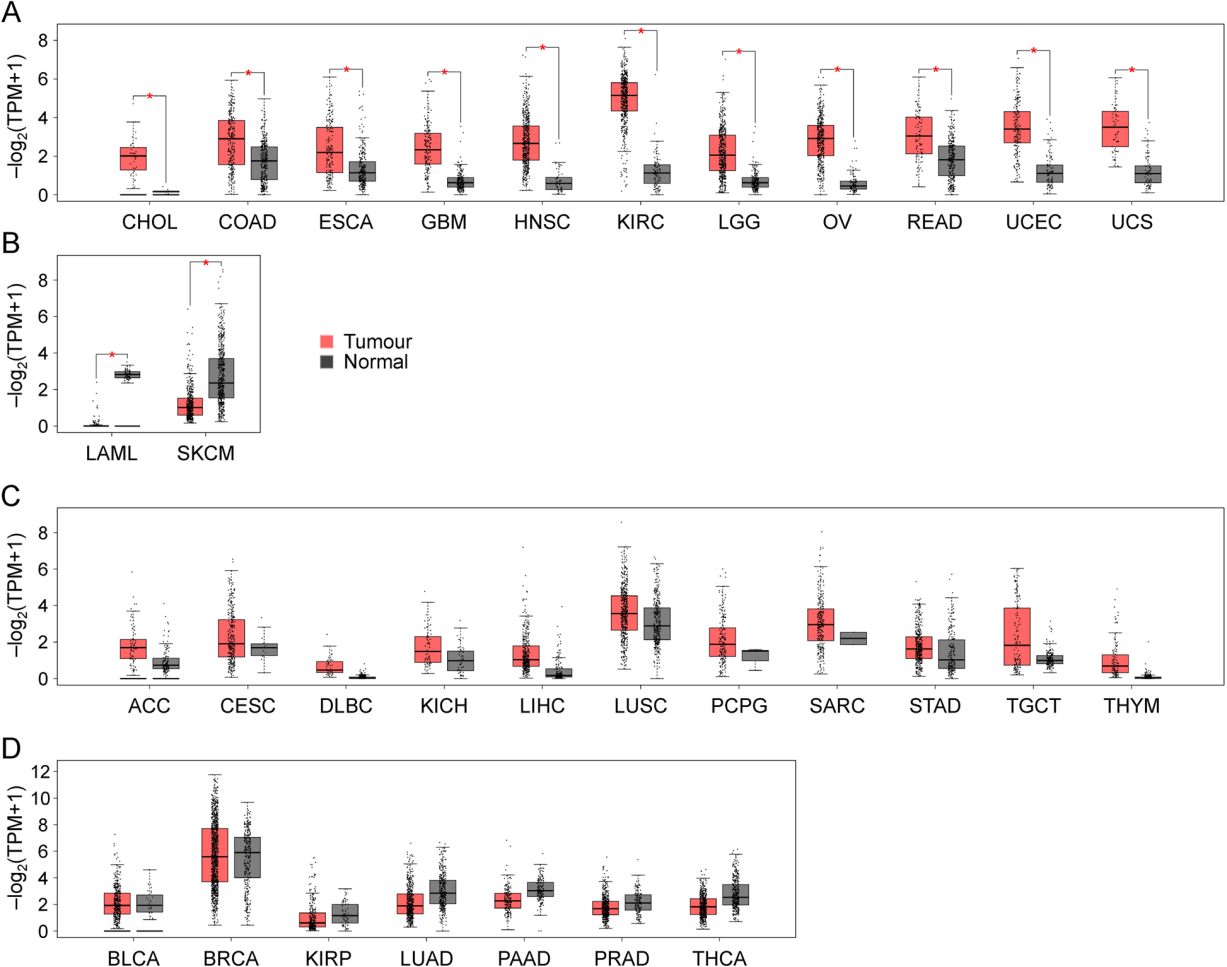
The comparison of STC2 expression between human tumours and their relative normal counterparts using the online tool (http://gepia2.cancer-pku.cn/). Panel A: CHOL, cholangiocarcinoma; COAD, colon adenocarcinoma; ESCA, esophageal carcinoma; GBM, glioblastoma multiforme; HNSC, head and neck squamous cell carcinoma; KIRC, kidney renal clear cell carcinoma; LGG, brain lower grade glioma; OV, ovarian serous cystadenocarcinoma; READ, rectum adenocarcinoma; UCEC, uterine corpus endometrial carcinoma; UCS, uterine carcinosarcoma. Panel B: LAML, acute Myeloid Leukemia; SKCM, skin cutaneous melanoma. Panel C: ACC, Adrenocortical carcinoma; CESC, Cervical squamous cell carcinoma and endocervical adenocarcinoma; DLBC, Lymphoid Neoplasm Diffuse Large B-cell Lymphoma; KICH, Kidney Chromophobe; LIHC, Liver hepatocellular carcinoma; LUSC, Lung squamous cell carcinoma; PCPG, Pheochromocytoma and Paraganglioma; SARC, Sarcoma; STAD, Stomach adenocarcinoma; TGCT, Testicular Germ Cell Tumours; THYM, Thymoma. Panel D: BLCA, Bladder Urothelial Carcinoma; BRCA, Breast invasive carcinoma; KIRP, Kidney renal papillary cell carcinoma; LUAD, Lung adenocarcinoma; PAAD, Pancreatic adenocarcinoma; PRAD, Prostate adenocarcinoma; THCA, Thyroid carcinoma. Red bar: tumour tissues; Gray bar: normal tissues. Red star indicates p values less than 0.05

Gene expression profiling and interactive analysis (GEPIA 2) [77] bioinformatic analyses further support the upregulation of STC2 in various human tumours compared to corresponding normal tissues, except for acute myeloid leukemia and skin cutaneous melanoma where STC2 expression is lower than their normal counterparts (Fig. 3). GEPIA 2 analysis indicates insignificant increase of STC2 expression in breast cancer comparing with their normal counterparts, showing inconsistence with the published wet-bench studies, which directly compared the expression of STC2 proteins in this tumour [50, 51]. Since the GEPIA2 bioinformatic analysis is generally based on mRNA levels, this inconsistence suggests the existence of complicated mechanisms to regulate STC2 expression at multiple levels.

Indeed, the expression of STC2 is regulated at both transcriptional and post-transcriptional levels. Interestingly, STC2 levels are induced under various stress conditions like endoplasmic reticulum (ER) stress, hypoxia and nutrient deprivation [78, 79]. Accordingly, STC2 can facilitate cells dealing with stress conditions and preventing cells from apoptosis. Functionally, STC2 has been reported to control a variety of cellular processes such as proliferation, migration and immune response. As such, it is hypothesized that STC2 is a critical contributor for the development of acquired resistance to chemo- and radio- therapies, thus holding the promise to be a universal therapeutic target for multiple types of cancers. This review focuses on discussing the regulation, biological importance and particularly, the potential clinical utilization of STC2 in the field of human cancers.

### STC2 is overexpressed in multiple types of human tumours

#### Breast cancer

Breast cancer, the second common cause of death for women, accounts for ~ 26% of all cancers diagnosed for women [80]. Most breast cancers (~ 70%) are positive for estrogen receptor (Er), a receptor interacting with circulatory estrogens. The constitutive activation of Er signaling leads to hyper-activation of downstream survival signaling that promotes cell growth and inhibits apoptosis. As such, anti-estrogen therapy becomes a standard treatment for patients with Er-positive breast cancers. Approximately 15–20% breast cancer patients demonstrate the amplification and/or overexpression of human epidermal growth factor receptor 2 (ErbB2) [81]. ErbB2 can form homodimers or heterodimers with other ErbB family members to activate downstream signaling that enhances the survival and proliferation of oncogenic cells; therefore, overexpression of ErbB2 is associated with poor prognosis in patients with breast cancer [82].

STC2 is co-expressed with Er in breast cancer cell lines and samples [29, 32, 50, 83, 84]. cDNA microarray revealed a threefold induction of STC2 mRNA upon estrogen treatment in breast cancer cells. Clinically, STC2 mRNA and protein levels are positively correlated with Er expression in human breast cancer specimens [29, 32, 84]. Although STC2 promoter contains canonical DNA-binding sites for Er, STC2 expression is indirectly controlled by Er signaling at the transcriptional level, which is mediated by other intermediary transcriptional factors [84]. Taken all analyzed breast cancer cohort into account, data show that patients with elevated STC2 levels have a better disease-free survival (DFS) rates [50, 51]. Interestingly, when it comes to hormone receptor-negative patients, STC2 levels are not associated with DSF rates [50]. These findings are supported by independent studies involving both woman and male breast cancer patients [53, 55]. It should be noted that the findings from another two studies don’t support the notion that STC2 could be applied to evaluate the prognosis of breast cancer patients [52, 54]. Therefore, the value and application of STC2 expression in the prognosis of breast cancers should be further evaluated based on the subtypes and other molecular characteristics.

### Colorectal cancer

Both mRNA and protein levels of STC2 are elevated in human colorectal cancer tissues comparing to the noncancerous counterparts [33, 56]. The genome-wise studies revealed higher STC2 mRNA levels in colorectal cancer than normal tissues [58, 59]. Clinically, increased STC2 expression is correlated with local invasion, lymphatic metastasis, tumour size and advanced disease stages. Patients with high STC2 expression showed poor prognosis comparing to those with low STC2 expression [33, 56]. Another study revealed STC2 can be induced in human colon cancer cells upon selenomethionine treatment, which is supposed to deprive intracellular cysteine or sulfhydryl sources [85]. While the biological roles of STC2 under this condition require further investigation, it is generally believed high level of STC2 expression predicts poor prognosis of colorectal cancer patients.

### Gastric cancer

Although its incidence is decreasing, gastric cancer remains one of the most common cancers worldwide. The gastric cancer screening is not a broadly-used routine test; thus, it is not uncommon that the majority of patients made their first visit with an advanced stage gastric cancer [86]. Postoperative adjuvant chemotherapy assists the improvement of patients’ prognosis with advanced stage tumours; therefore, it’s beneficial to identify novel biomarkers of gastric cancer to assess the necessity to take adjuvant therapy [62]. cDNA microarray revealed elevated STC2 levels in gastric cancer comparing to normal tissues. Consistently, STC2 levels are higher in gastric cancer tissues than those in normal mucosa; elevated STC2 expression is correlated with tumour invasion, lymphatic metastasis and poor prognosis [62, 63]. Furthermore, blood STC2 levels have been shown to be a useful biomarker to screen and evaluate the prognosis of patients with gastric cancer [87, 88].

### Esophageal squamous cell carcinoma (ESCC)

In a study looking for genes related to ESCC progression, STC2 is illustrated to be upregulated in ESCC comparing to normal tissues [35]. Ectopic expression of STC2 enhances the proliferation and invasiveness of ESCC cells; while knockdown of STC2 compromises the proliferation of ESCC cells with high STC2 levels [35]. Further clinicopathological analyses demonstrated STC2 levels are correlated with lymphatic invasion, lymph node metastasis, and poor prognosis in ESCC patients, suggesting STC2 is a potential biomarker and a therapeutic target for ESCC.

### Prostate cancer

Prostate cancer is one of the major public health problems and the second leading cause of cancer-related death for men. In 2021, the estimated incidence will be 248,530 cases and 34,130 men will probably die of prostate cancer [89]. Currently, surgery and radiotherapy remain the most effective treatment for patients with local tumours; however, approximately 30 percent of patients suffer from relapse even after treatment [90]. The majority of relapsed patients still demonstrate acceptable response to medical androgen ablation or castration. Unfortunately, the tumours will finally become castration-resistant and more aggressive. Therefore, it’s with great importance to search for new therapeutic candidates in order to treat patients with castration-resistant prostate cancer (CRPC) [36].

CRPC cells show increased STC2 mRNA levels relative to those from castration-naïve prostate cancer cells. Clinically, the expression of STC2 is correlated with high Gleason scores [36]. Knockdown of STC2 compromises the colony formation; while ectopic expression of STC2 promotes cell proliferation [36], indicating STC2 may serve as a novel diagnostic biomarker for aggressive prostate cancer and a new therapeutic target for CRPC. In fact, STC2 is among the most upregulated genes in hormone insensitive prostate cancer cells treated with pigment epithelium-derived factor, but the roles of STC2 in CRPC and its potential as a therapeutic target remain unclear and require further investigation [91].

### Renal cell carcinoma (RCC)

RCC represents the most common kidney tumours in adults. Approximately 30% RCC patients are diagnosed with metastatic lesions at first visit; some patients will finally develop metastasis even with the removal of local tumours [92]. There remains no effective treatment for patients with metastatic RCC; therefore, it’s critical to search for novel therapeutic targets. Two independent whole genome-wise studies identified STC2 as one of the most upregulated genes in RCC tumour tissues relative to adjacent normal kidney [93, 94]. Immunohistochemical (IHC) staining demonstrated that STC2 is mainly expressed in distal tubuli and glomeruli in normal kidney tissues with both cytoplasmic and membranous signals detected in RCC tumours. Both mRNA and protein levels of STC2 are higher in RCC than in non-tumorous adjacent tissues [37]. The expression of STC2 is correlated with the aggressiveness of primary RCC and the survival rates post radical nephrectomy [37]. Since STC2 is a secreted protein, its serum levels can be applied as a biomarker to evaluate the progression of RCC, but further investigations are required to clarify this probability.

### Other tumours

In addition to the above-mentioned tumours, STC2 is also upregulated in other tumours like nasopharyngeal carcinoma, HNSCC, HCC, osteosarcoma, ovarian cancer, lung cancer, pancreatic cancer, endometrial cancer, cervical cancer, neuroblastoma, gallbladder cancer and cholangiocarcinoma as listed in Table 1. In brief, elevated STC2 levels are correlated with tumour progression, disease stage and patients’ prognosis. STC2 is detected as a secreted protein in the extracellular proteome of HCC cells [95]; in the absence of cysteine, STC2 is upregulated, highlighting that as a stress response factor STC2 may reduce cell proliferation when nutrient is limited [96]. The expression of STC2 in vascular cells is correlated with decreased DFS in ovarian cancer [42]. STC2 is identified as a screening marker for endometrial cancer in combination with CRELD1, GRK5 and SLC25A27 [97]. In human neuroblastoma, the expression of STC2 is associated with clinical stages [47]. Moreover, in human fibrosarcoma cells, STC2 has been reported to be phosphorylated between Serine-285 and Serine-298 in the C-terminal region by casein kinase [98].

Taken together, the above findings show that increased expression of STC2 is negatively correlated with patients’ prognosis for most human cancers except breast cancer [29, 32, 84], which might be a result of different tissue origins and specific genetic context, such as the existence of complicated hormone-related mechanisms. As a secreted glycoprotein, STC2 has been detected in the sera of patients with colorectal cancer [33], gastric cancer [87], and laryngeal cancer [64]. In accordance, STC2 is also detected in the culture medium of tumour cell lines derived from glioma and fibrosarcoma [98]. Therefore, it is promising to systematically investigate the clinicopathological importance of serum STC2 levels in tumor diagnosis and in evaluating patients’ prognosis for multiple types of human cancers.

### Transcription factors involved in STC2 regulation

#### Activating transcription factor 4 (ATF4)

**Fig. 4:**
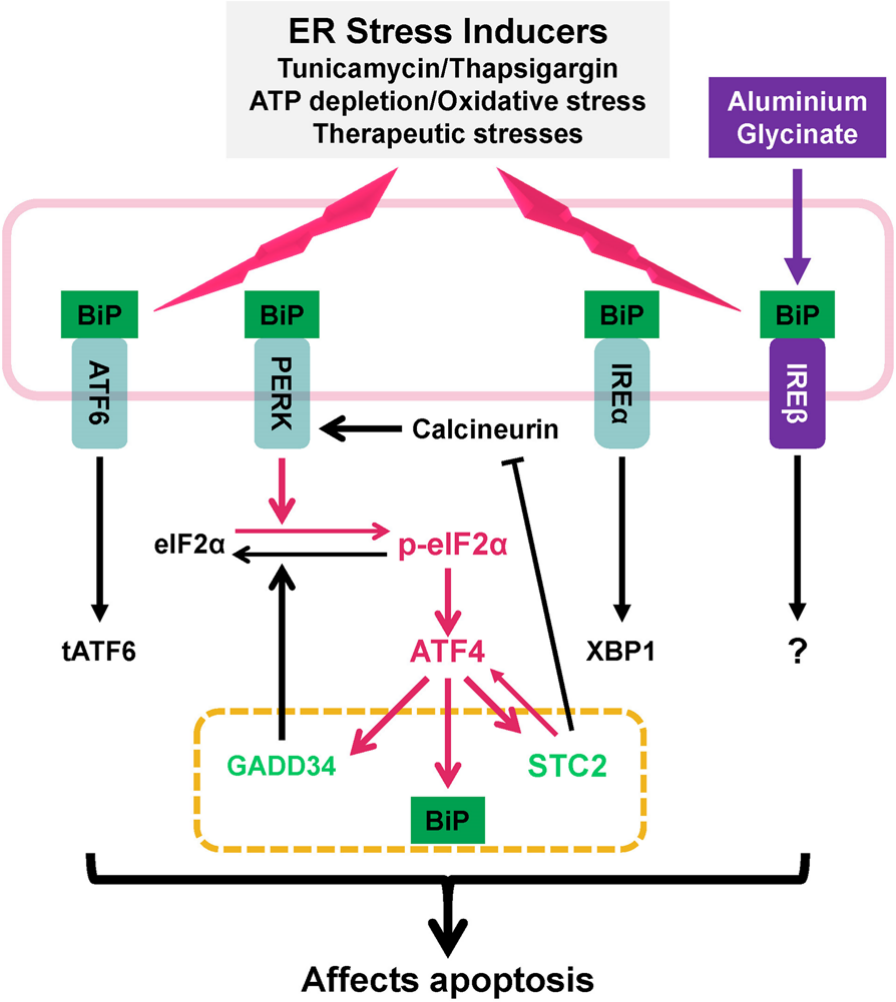
STC2 modulates the severity of ER stress response, promotes cell survival and prevents apoptosis. Hypoxia, nutrient depletion associated ATP depletion, and oxidative stress trigger ER stress. Similarly, therapeutic treatment and some compounds disrupting ER functions also trigger ER stress. Under such conditions, BiP, the negative regulator of ATF6, PERK and IRE1, is sequestrated by misfolded proteins accumulated in the ER lumen. The activation of ATF6 (tATF6, truncated and activated form), ATF4 and XBP1 leads to transcriptional upregulation of genes involved in ER homeostasis, ER biogenesis, inflammatory response, protein folding and degradation. Severe and prolonged ER stress leads to the activation of JNK/NF-κB and CHOP pathways which promote apoptosis. Under moderate stress conditions, the p-eIF2α-ATF4 axis serves as a modulator of the severity of ER stress response by activating three down stress genes: 1) as the negative regulator of ER stress, BiP upregulation facilitates restoring ER homeostasis; 2) GADD34, a phosphoprotein phosphatase, directly dephosphorylates eIF2α to attenuate ATF4-mediated stress response; and 3) importantly, STC2 performs its function through affecting ATF4 or calcineurin, a calcium and calmodulin dependent serine/threonine protein phosphatase that is evidenced by the report showing aluminium toxicity causes ER stress, which activates IRE1β, but not ATF4, finally leading to apoptosis

ATF4 plays a critical role in mediating integrated stress response (IRS), and ATF4 is correlated with STC2 expression. Tumour microenvironment is characterized by uneven oxygen levels, insufficient nutrient supply and low pH [99]; these stress conditions lead to ER stress that disrupts protein maturation, accumulates unfolded or misfolded proteins, and ultimately activates the evolutionarily conserved response, unfold protein response (UPR) [100]. Several factors, such as PKR-like ER kinase (PERK), inositol-requiring protein 1 (IRE1) and activating transcription factor 6 (ATF6) can be activated through the loss of interaction with Bip and/or the direct binding with unfolded or misfolded proteins [101]. UPR regulates apoptosis and several other pathways to control cell survival [102]. Besides the well-characterized UPR genes, factors involved in cellular Ca^2+^ homeostasis, such as Nucleobindin 2, sarco- (endo-) plasmic reticulum Ca^2+^-ATPase 2, calcitonin gene-related peptide 2 and STC2 can be induced upon the treatment with either tunicamycin (an inhibitor of N-linked glycosylation) or thapsigargin (an inhibitor of sarco- (endo-) plasmic reticulum Ca^2+^-ATPase) (Fig. 4) [103]. Both tunicamycin and thapsigargin have been shown to upregulate the expression and secretion of STC2 in a time-dependent manner. The expression of STC2 is enhanced by ER stress inducers like dithiothreitol, methyl methanesulfonate, hydrogen peroxide (H_2_O_2_) and hypoxia but not by non-ER stress inducers such as high extracellular Ca^2+^ levels, high osmolarity, ultraviolet radiation, staurosporine or heat shock [103], suggesting that STC2 is likely a direct downstream target of UPR or ER stress. Mechanistically, ER stress-induced STC2 expression depends on ATF4 pathway but not XBP-1 and ATF6.

On the other hand, STC2 can also modulate ATF4 activity and function as an inhibitor of apoptosis. The conserved response upon moderate ER stress facilitates cellular adaptation to enhance survival, for example, induction of ATF4 and its downstream genes, BiP and GADD34, specifically ameliorates and limits the severity of ER stress. Upon persisting and severe ER stress, the three ER stress-triggered responsive arms ATF4, ATF6 and IRE1 may cooperatively trigger apoptosis via CHOP and JNK/NF-κB pathways. As a cytotoxic reagent, aluminium induces apoptosis through activating ER stress. Unlike tunicamycin, aluminium glycinate induces the expression of IRE1β instead of IRE1α, and it suppresses the transcription of other UPR genes like molecular chaperone BiP/Grp78, Ca^2+^-binding chaperones (calnexin and calreticulin) and STC2 (Fig. 4) [104]. In addition, STC2 compromises calcineurin expression and calcineurin-dependent PERK phosphorylation in cerulein-induced pancreatitis mouse model [105]. Although PERK activity is suppressed, ATF4 expression is upregulated in STC2 transgenic mice, suggesting a feed-forward model of STC2 in regulating ATF4-dependent adaptive pathway through unknown mechanisms. In ischemic rat brain, subsequent reperfusion induces STC2 expression in brain tissues previously exposed to hypoxia. Functionally, STC2 enhances cell survival upon exposure to reperfusion associated oxidative stress, which is a common cause of ER stress as well. Therefore, STC2 demonstrates protective effects through limiting the severity of ER stress via modulating ATF4 signaling.

Besides PERK, there are three other stress-activated kinases GCN2, PKR and HRI, that can activate the eIF2α-ATF4 axis and facilitate cellular homeostasis and adaptation to stress conditions such as proteotoxicity, nutrient withdrawal, oxidative stress and viral infection [106–108]. Under such conditions, phosphorylated eIF2α inhibits global protein translation, but selectively promotes the translation of proteins such as ATF4 that are critical for stress response and cell survival. Interestingly, previous cDNA microarray screening identified STC2 is also upregulated upon either glutamine- or glucose- deprivation, both conditions can trigger ER stress via different mechanisms [78, 109]. For example, glutamine deprivation promotes the phosphorylation of eIF2α through amino acids deprivation triggered GCN2 activation. Glucose deprivation triggers ER stress through the following mechanisms: 1) glucose deprivation impairs ATP production; 2) inhibited pentose pathway causes intracellular redox imbalance, leading to oxidative stress; and 3) low glucose also suppresses protein glycosylation, resulting in protein unfolding/misfolding. Further studies are required to dissect how STC2 expression is controlled in cells under various stress conditions, and how it regulates biological processes in stressed cells. Nevertheless, ATF4-dependent induction of STC2 expression establishes the principle that STC2 is a general stress-responsive factor mediating the adaptation of cells to various types of stresses.

### Hypoxia-inducible factor-1 (HIF-1)

Mammalian cells require sufficient and constitutive oxygen supply for a variety of metabolic processes [110–112]. Hypoxia is a common phenomenon in solid tumours, under which the proliferation of tumour cells usually outgrows the growth of vascular vessels, leading to insufficient oxygen supply [100]. Under hypoxic conditions, tumour cells activate the evolutionarily conserved hypoxic response through stabilizing HIF-1α that forms a HIF-1 heterodimer through interacting with HIF-1β [113]. Then, HIF-1 binds to hypoxia-response element (HRE) to enhance the expression of angiogenic, glycolytic and other relevant genes that mediate the adaptive response [114].

**Fig. 5:**
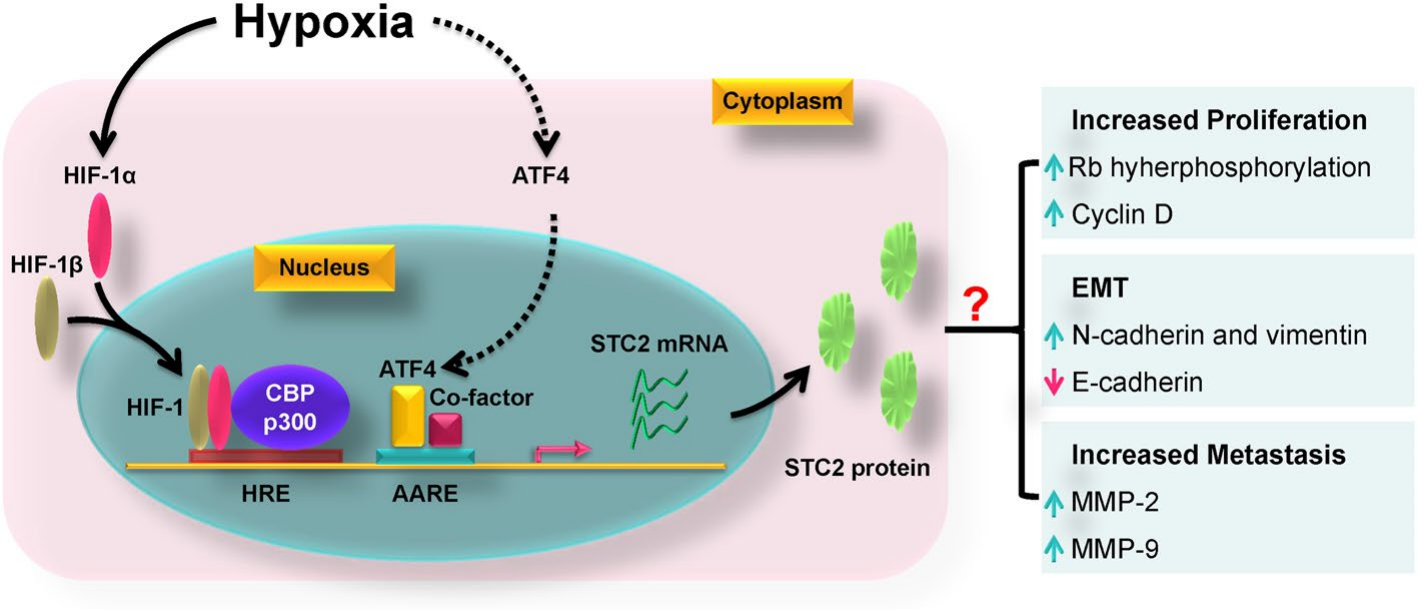
The biological functions of STC2 under hypoxic conditions. STC2 promoter contains hypoxia response elements (HREs) and amino acid response elements (AAREs). Under hypoxic conditions, HIF-1α is stabilized, and forms the HIF-1 heterodimer through interacting with HIF-1β. Then, HIF-1 is translocated into the nucleus where it binds to CBP/p300 and form a transcription complex to induce the expression of STC2. In addition, hypoxia also induces the expression of ATF4 through increased gene transcription, protein translation and protein stabilization. Thereafter, ATF4 is translocated into nucleus and form a transcriptional complex with co-factors to enhance the expression of downstream genes; however, it remains elusive whether ATF4 also contributes to STC2 upregulation under hypoxic conditions. Functionally, STC2 promotes cell cycle progression through enhancing cyclin D expression and Rb phosphorylation; STC2 induces epithelial-mesenchymal transition (EMT) through upregulating mesenchymal markers (N-cadherin and vimentin) and downregulating E-cadherin; in addition, STC2 can drive cell migration and invasion through the upregulation of matrix metalloproteinase (MMP) -2 and -9

cDNA microarray analysis demonstrated hypoxia induces the expression of STC2 in proximal tubular epithelial cells; the longer the cells expose to hypoxia, the higher STC2 mRNA levels are detected [115]. Bioinformatic analysis reveals STC2 promoter contains conserved putative HRE binding motifs; consistently, electrophoretic mobility shift assay demonstrates HIF-1 directly binds to those motifs (Fig. 5), and the constitutively active form of HIF-1α (AdCA5) also upregulates STC2 expression at both mRNA and protein levels. There are several CpG islands in the 5’-flanking region of the upstream sequence relative to STC2 translation start site; the methylation of these CpG islands compromises STC2 expression in cells derived from human ovarian cancer, pancreatic cancer, colon adenoma and leukemia, but not in normal epithelial cells [116]; furthermore, 5-aza-2′-deoxycytidine, a DNA methylation inhibitor successfully induces STC2 expression [116]. In addition, chetomin, a p300 inhibitor, also reverses hypoxia-induced STC2 expression [117], highlighting CBP/p300 are indispensable co-activators of HIF-1 for STC2 upregulation. As a direct target of HIF-1, STC2 is proposed to facilitate cell survival, proliferation and migration when exposed to low oxygen conditions [103, 116–118].

### High-mobility gene group A2 (HMGA2)

High Mobility Group A (HMGA) proteins belong to the family of non-histone chromatin factors that possess transcriptional activities [119]. HMGA family has three members: HMGA1a, HMGA1b and HMGA2, all three contain a highly conserved DNA binding domain and a negative charged c-terminal tail. HMGA2 regulates cell growth, differentiation and migration [120]. HMGA2 is expressed in embryonic tissues and certain malignant tumours like ovarian cancer but not normal adult tissues. In a study aiming to understand the role of HMGA2 in regulating tumour development and progression, cDNA microarray was applied to investigate the mRNA expression profile of ovarian surface epithelial cells with various levels of HMGA2. Among thirty-six upregulated genes, STC2 is identified [121], suggesting the regulation of STC2 by HMGA2. Another independent study further illustrated the correlation between HMGA2 and STC2 in human high-grade serous cancer samples. Bioinformatic analysis demonstrated putative HMGA2 binding sites in the 5’ transcription start site of STC2 promoter; moreover, luciferase reporter assay identified ectopic HMGA2 expression can enhance the luciferase activity mediated by STC2 promoter in a dose-dependent manner, suggesting STC2 is transcriptionally induced by HMGA2 [76]. Consistently, a positive correlation between HMGA2 and STC2 IHC staining score is also revealed in human epithelial ovarian cancer samples. These findings provide evidence that HMGA2, as an oncofetal protein, can transcriptionally upregulate STC2 during fetal development and oncogenesis, suggesting that STC2 plays important roles in those processes.

### Aryl hydrocarbon receptor (AhR)

AhR, a transcription factor with multiple functions, works as an environmental sensor that facilitates the integration of different signaling pathways. Under physiological conditions, AhR involves in many processes like defending chemicals and pathogens, development, immune response and reproduction, thus facilitating cell adaptation to environmental stresses. cDNA microarray found STC2 as a putative downstream target of AhR in primary human hepatocytes and HepaRG cells [122, 123]. Bioinformatic analysis demonstrated AhR can be recruited to the potential xenobiotic response elements in STC2 promoter that is evidenced by chromatin immunoprecipitation; moreover, AhR agonists effectively block STC2 expression, indicating STC2 is an AhR-dependent gene. Mechanistically, upon the treatment of cinnabarinic acid (the AhR agonist), metastasis-associated protein 2 is recruited to the STC2 promoter, leading to the acetylation of lysine residue in histone H4 and the transcription of STC2 gene [124]. Biologically, STC2 protects cells from various stress conditions like ER stress induced by H_2_O_2_, thapsigargin and ethanol [124, 125]. The induction of STC2 by AhR indicates a role of STC2 in cellular response to environmental hazardous agents.

### Specificity protein 1 (Sp1)

Sp1, a transactivation molecule, belongs to Sp or Krüppel-like factor protein family [126]. Sp1 functions to promote the activities of oncogenes for cell survival, tumour progression and metastasis. Structurally, Sp1 contains N-terminal inhibitory domain, Transactivating domain, A highly charged group of 69 residues, DNA binding domain and C-terminal domain for synergistic transactivation. Sp1 is upregulated in several human cancers like colorectal cancer, and the expression of Sp1 is correlated with poor prognosis [127]. Bioinformatic analyses revealed potential Sp1 binding sites in STC2 promoter. In accordance, luciferase assay demonstrated genetic manipulation of Sp1 alters STC2 promoter-mediated luciferase activity in a dose-dependent manner in colorectal cancer cells. Either ectopic expression or knockdown of Sp1 can alter STC2 protein levels [128], further denoting the direct regulation of STC2 by Sp1. Considering Sp1 as a general transcription factor commonly involved in the basal level expression of many genes, it is likely a necessary but insufficient transcription factor for stress-induced STC2 transcription.

### Non-coding RNAs-mediated post-transcriptional regulation

The non-coding RNAs (ncRNAs) define a group of RNAs that are not translated into proteins [129]. The ncRNAs can be classified into two major groups according to their sizes: those with length shorter than 200 nt including microRNA (miRNA), small interfering RNA (siRNA), small nuclear RNA (snRNA), and piwi-interacting RNA (piRNA); and those with length longer than 200 nt, such as long non-coding RNAs (lncRNAs) [130]. miRNAs are small, single-stranded, and about 22 nucleotides in length [131, 132]. The sequences encoding miRNAs are mainly located within protein-coding genes, and they share the promoters with protein-coding genes; in other conditions, miRNAs are located in the intergenic regions [131]. Mechanistically, miRNAs recognize and bind to their targets through the complementary sequence within the targeted mRNAs. In general, a specific miRNA can target more than one mRNA, and a specific mRNA can be targeted by several different miRNAs. Biologically, miRNAs mediate the post-transcriptional gene repression or mRNA degradation [132], and they can regulate cell differentiation, maturation, proliferation, survival and immune response that are related to tumorigenesis, tumour metastasis and progression [133]. Functionally, lncRNAs perform their functions through transcriptional regulation in either *cis* or *trans* manner. In addition, lncRNAs can function as molecular scaffolds that facilitate the interaction between mRNAs, miRNAs, DNAs and their relevant proteins like transcription factors, RNA-binding proteins and chromatin modifying complexes [134]. Therefore, lncRNAs are essential for various biological processes, and the dysregulation of lncRNAs presents in many pathological conditions, particularly, cancers [135]. The following are some examples of miRNAs and lncRNAs reported to regulate STC2 expression.

### *miR-206 regulates STC2 in gastric cancer and HNSCC*

The miR-206 is located in the human chromosome 6p12.2. It is highly expressed in muscle tissues and controls the process of myogenesis. miR-206 demonstrates tumour-suppressor activities during tumour development and progression in breast cancer, lung cancer and rhabdomyosarcoma [136]. In gastric cancer, increased miR-206 levels are inversely correlated with tumour invasion, metastasis and disease stage. Bioinformatic analysis plus overexpression experiments revealed STC2 is a potential target of miR-206 [137]. In accordance, miR-206 expression is negatively associated with tumour stage in HNSCC. On the other hand, increased LINC00460 can effectively antagonize miR-206 function and upregulate the expression of STC2, which inhibits autophagy through downregulating Beclin-1 and suppresses apoptosis by activating Erk and Akt signaling [138]. Moreover, another long non-coding RNA, HOTAIR, also functions as a sponge to antagonize miR-206 activity, therefore, inducing STC2 expression to enhance cell proliferation, migration and invasion through activating PI3K-Akt signaling in HNSCC [139].

### Other microRNAs

Other microRNAs are also found to regulate STC2 expression in human cancers. miR-485-5p is significantly downregulated in human HCC samples when comparing to normal liver tissues, and its expression is correlated with tumour size and tumour number. Bioinformatic algorithm prediction suggests STC2 is a target of miR-485-5p. Genetic manipulation of miR-485-5p alters luciferase reporter activity-mediated by the 3’-untranslated region (UTR) of STC2 mRNA and STC2 protein levels in HCC cells [140]. Another study illustrated a reverse correlation between miR-184 and STC2 mRNA levels in human glioblastoma tissues. A complimentary sequence of miR-184 was identified in the 3’-UTR of STC2 mRNA. Further analyses demonstrated that miR-184 can downregulate STC2 expression through a direct interaction with the 3’-UTR of STC2 [141]. Functionally, miR-184 suppresses the proliferation, invasion and migration of glioblastoma cells in an STC2-dependent manner. A binding sequence of miR-190 was found in the 3’-UTR of STC2 mRNA and the luciferase reporter assay showed a direct regulation of STC2 by miR-190 [142]. miR-190 regulates epithelial-mesenchymal transition (EMT) and angiogenesis through altering the activity of Erk and Akt signaling in an STC2-dependent manner in breast cancer cells. Furthermore, miR-381 inhibits cell proliferation, migration and invasion in HNSCC cells. Bioinformatic analysis and dual luciferase reporter assay highlight STC2 as a direct target of miR-381 [143]. Importantly, ectopic expression of STC2 antagonizes the inhibiting activity of miR-381 in HNSCC cells through regulating the FAK-PI3K-Akt-mTOR signaling pathway.

### LncRNAs

LINC00460 stands for the long intergenic nonprotein coding RNA 460 that is located in Chromosome 13q33.2 region with a length of 935 nt [144]. LINC00460 is upregulated in a variety of human cancers and demonstrates oncogenic properties. Particularly, LINC00460 is upregulated to drive the development of HNSCC, colorectal cancer, RCC, HCC, lung cancer, thyroid cancer, glioma, meningioma, etc. [145]. Clinically, the expression of LINC00460 is correlated with lymph node metastasis, EMT and patients’ prognosis [146]. There is elevated expression of LINC00460 and STC2 in human HNSCC while with a clear reduction of miR-206 levels [138]. Mechanistically, LINC00460 works as a sponge to compromise miR-206 function, leading to the upregulation of STC2 at both mRNA and protein levels, and finally the activation of Akt signaling that suppresses apoptosis and autophagy [138]. Consistently, lncRNA homeobox transcript antisense RNA (HOTAIR) is overexpressed in human HNSCC samples, and it can also promote STC2 expression through functioning as a sponge of miR-206 [147]. In addition, other two lncRNAs are reported to regulate STC2 expression, for example, ROR1-AS1 functions to sponge miR-670-3p that suppresses STC2 expression in cervical cancer [148]; SNHG17 promotes STC2 expression through antagonizing the activity of miR-361-3p in rectal cancer [149].

The discovery of non-coding RNA-mediated regulation of STC2 expression provides new insights into the regulation of STC2, and opens new doors for therapeutical approaches to target STC2.

### The biological roles of STC2 in human cancers

#### STC2 promotes tumour cell survival and tumour growth

Tumour growth depends on the proliferation of tumour cells and the ability of tumour cells resisting cell death. In general, STC2 drives cell proliferation and maintains cell survival in a plethora of tumour cells, particularly for those under stress conditions [33, 35, 36, 47, 62, 84, 116–118]. Knockdown of STC2 leads to apoptosis upon treatment with thapsigargin but not tunicamycin or staurosporine; on another hand, overexpression of STC2 successfully inhibits apoptosis-induced by thapsigargin [103]. In transgenic mice, STC2 protects cells from necrosis and apoptosis in a pancreatitis model-induced by cerulein [105]. Under hypoxic conditions, STC2 is upregulated in SKOV3 and MCF-7 cells, leading to cyclin D induction and Rb hyper-phosphorylation, finally driving cell proliferation (Table 2) [117]. In accordance, knockdown of STC2 compromises colony formation of PC-3 cells; while overexpression of STC2 promotes 22Rv1 cell proliferation [36]. Moreover, STC2 also promotes the proliferation of tumour cells derived from ESCC [35], gastric cancer [62], colorectal cancer [33], nasopharyngeal carcinoma [79], etc.. Elevated STC2 levels are correlated with tumour size in colorectal cancer [33]; chorioallantoic membrane of chicken embryo assay revealed ectopic expression of STC2 enhances the growth of tumour xenografts, resulting in tumours with bigger size than control group [47]. However, overexpression of STC2 compromises cell proliferation as well as colony formation in breast cancer cells [84], suggesting STC2 possesses tumour suppressor-like activities in breast cancer. These findings indicate STC2 performs its roles in a context-dependent manner. Further studies are required to dissect the detailed molecular mechanisms in order to develop new therapies.

**Table 2:** Reported biological functions of STC2 in human cancers

Biological functions	Involved processes	Mechanisms	References
Cell proliferation	Promotes cell cycle progression	Induces cyclin D expression and Rb hyper-phosphorylation	[117]
Cell survival	Suppresses apoptosis	Activates MAPK signaling Activates PI3K-Akt signaling Activates the Jun-Axl-Erk signaling	[43, 150, 151]
Tumour metastasis	Cell migration and invasion	Promotes the production of MMPs Drives EMT through inducing N-cadherin and vimentin and suppressing E-cadherin	[47, 118]
Stress Response	Integrated stress response as a result of unfolded/misfolded proteins or the deprivation of nutrients	Compromises calcineurin expression and calcineurin-dependent PERK phosphorylation Upregulates ATF4 expression in STC2 transgenic mice	[105]
Immune avoidance	Functions as a metagene Compromises the activity of IFN-γ-producing CD8 + Tc1 cells	Reduces the expression of immune-associated metagenes Modulates the immunomodulatory properties of mesenchymal stem cells	[52, 152]

### STC2 facilitates tumour metastasis

Tumour metastasis involves four major steps: 1) acquisition of a metastatic phenotype, tumour cells undergo EMT through downregulating epithelial markers and upregulating mesenchymal markers, leading to reduced cell–cell adhesion, apical-basolateral polarity and increased motility [153]; 2) breakdown of the extracellular matrix (ECM) by matrix metalloproteinase (MMP) -2 and -9 secreted by tumour cells [154]; 3) intravasation, tumour cells migrate through the perivascular tissue, penetrate the blood vessel walls and become circulating tumour cells (CTCs) [155]; 4) extravasation and formation of metastatic tumours, CTCs invade through the blood vessel walls and reside in distal tissues or organs and finally form a metastatic tumour [156]. STC2 enhances tumour metastasis through promoting EMT and upregulating MMP -2 and -9 [47, 118]. Under hypoxic conditions, HIF-1 promotes the expression of STC2 to upregulate mesenchymal markers like N-cadherin and vimentin while to suppress the expression of E-cadherin, an epithelial marker; moreover, ectopic expression of STC2 upregulate MMP-2 and -9 that facilitate the degradation of ECM (Table 2) [118]. Transwell assay revealed overexpression of STC2 enhances the invasion of ovarian cancer cells through matrigel or HUVEC -coated wells [118]; in accordance, overexpression of STC2 upregulates MMP-2 expression and enhances the invasive ability of neuroblastoma cells [47]. Furthermore, elevated STC2 levels are associated with invasiveness and metastasis in human tumours like ESCC [35], gastric cancer [62], colorectal cancer [33], RCC [37] and neuroblastoma [47].

### STC2 regulates cellular response to nutrient-deprivation

Metabolic reprogramming is regarded as an emerging hallmark of human cancers [157]. Generally, there are two major characteristics of cancer metabolism: Warburg effect and active glutaminolysis [110, 111, 158], which define the unique metabolism of glucose and glutamine, respectively. Warburg effect emphasizes tumour cells can utilize glucose through glycolysis even in the presence of oxygen, and glutaminolysis indicates the conversion of glutamine to glutamate that is catalyzed by glutaminase [159]. Glucose and glutamine can not only provide energy for tumour cells, but also can be employed to produce building blocks for proliferating cells and factors to resist damages under stress conditions; therefore, it is important and useful to dissect the molecular scenario how tumour cells response to nutrient-deprived stresses and maintain cellular homeostasis in order to search for potential therapeutic candidates [78]. According to this hypothesis, we performed cDNA microarray analysis to investigate the gene expression of Hep3B cells upon glucose- or glutamine- deprivation relative to that of Hep3B cells in complete medium [120]. Importantly, STC2 is identified as one of the most upregulated genes upon either glucose- or glutamine- deprivation. In another study of our laboratory, we found glucose-deprivation can induce STC2 at protein levels in CNE2 nasopharyngeal carcinoma cells [79]. Additionally, other independent studies also found the induction of STC2 upon cysteine-deprivation or selenomethionine treatment that deprives intracellular sulfhydryl groups hence triggering oxidative stress [85, 96]. Taken together, these studies reveal that STC2 is a common responding factor that is induced by nutrient deprivation (Table 2).

Recently, a study with bioinformatic analysis found STC2 can be grouped with glycolytic genes to assess the prognosis of patients with osteosarcoma [41]. Mechanistically, STC2 promotes glucose utilization though inducing the expression of glycolytic genes including Glucose Transporter 1 and Lactate Dehydrogenase A [41]. Currently, our laboratory is working on the detailed molecular mechanisms of how STC2 is induced upon glutamine-deprivation and its biological functions in metabolic reprogramming during tumorigenesis and tumour progression.

### STC2 promotes tumour immune avoidance

Many studies demonstrate it is promising to evaluate the therapeutic efficacy of adjuvant chemotherapy through detecting the presence of pre-existing or treatment-induced antitumor immune response, as the immune response will facilitate the development of therapeutic vaccines by directly converting tumour tissues to antigens if the chemotherapies induce immunogenic cell death. Mechanistically, immunogenic cell death is characterized by ER stress and autophagy that regulates the expression of metagenes (the immune-relevant transcripts or gene sets). STC2, overexpressed in T cells with a Th2 response, is identified as a metagene that demonstrates negative correlation with immune-associated metagenes in breast cancer [52]. Moreover, STC2 can modulate the immunomodulatory properties of mesenchymal stem cells, which compromise the activity of IFN-γ-producing CD8^+^ Tc1 cells through interacting and altering the activity of heme oxygenase 1 (Table 2) [152]. The plasma-protein histidine-rich glycoprotein (HRG), a cation- and heparin- binding plasma glycoprotein, controls the phenotypic switching of macrophages through enhancing cytokine production and phagocytotic activity in tumours, resulting in the vessel normalization and antitumor immune responses. Unbiased analysis identified as a binding partner of HRG, STC2 can modulate the immune response of inflammatory cells [160]; however, the in vivo biological importance of STC2-HRG complex remains to be further dissected.

### Roles of STC2 in mediating tumour resistance to therapies

#### STC2-mediated resistance to chemotherapy

STC2 is upregulated upon exposure to several chemotherapeutic treatments, such as, anti-vascular endothelial growth factor (VEGF) antibody, cisplatin, oxaliplatin and tyrosine kinase inhibitors (TKIs), highlighting its potential in mediating the development of acquired resistance to chemotherapies (Table 3).

**Table 3:** STC2 mediates tumour cells’ resistance to therapies

Treatments	Tumours	Drug	Biological functions of STC2	References
Chemotherapy				
	Colorectal cancer	Bevacizumab (Anti-VEGF)	Promotes cell proliferation and migration due to reduced oxygen levels	[161]
	Cervical cancer	Cisplatin	Promotes cell proliferation Facilitates the activation of MAPK signaling pathway	[150]
	Colorectal cancer	Oxaliplatin	Compromises apoptosis Activates the PI3K-Akt signaling to upregulate P-glycoprotein	[151]
	HCC	Paclitaxel	Upregulates the expression of pro-survival proteins like P-glycoprotein and Bcl-2 that enhance cell viability	[162]
	Lung adenocarcinoma	EGFR TKI	Activates the Jun-Axl-Erk signaling to enhance cell survival	[43]
Radiotherapy				
	Nasopharyngeal carcinoma	‒	Promotes colony formation Suppresses apoptosis Drives cell cycle progression from G_1_ to S phase Enhances migratory and invasive ability of tumour cells	[38, 79]

The application of bevacizumab, the anti-VEGF antibody, successfully inhibits the growth of human colon cancer xenografts through blocking cell cycle progression at early stage and inducing apoptosis at late stage. cDNA microarray revealed bevacizumab induces STC2 expression through activating HIF-1 signaling in orthotopic human colon cancer xenografts [161]. Since STC2 drives cell proliferation and migration under hypoxic conditions; it is not surprised that STC2 is overexpressed in liver metastatic tumours of human colorectal carcinoma treated with bevacizumab relative to those without treatment, indicating STC2 may contribute to the development of acquired resistance to anti-VEGF treatment.

Cisplatin-resistant cervical cancer cells demonstrate increased STC2 mRNA and protein levels, while knockdown of STC2 effectively compromise cell proliferation and induce apoptosis in resistant cells [150], indicating STC2 as a mediator of tumour cells’ adaptation to cisplatin and it can be targeted to overcome the acquired resistance. Mechanistically, resistant cells demonstrate increased phosphorylation of Erk, JNK and p38, which can be reversed by STC2 knockdown. In accordance, STC2 is upregulated in human colorectal cancer cells with resistance to oxaliplatin relative to their parental counterparts. Moreover, increased STC2 protein levels were observed in the culture medium from resistant cells. Therapeutically, knockdown of STC2 effectively re-sensitizes resistant cells to oxaliplatin, and exposure of cells to recombinant STC2 protein is sufficient to compromise apoptosis induced by oxaliplatin in parental colorectal cancer cells [151], demonstrating STC2 performs its biological functions at least partially through a paracrine mechanism. Mechanistic analyses indicate that STC2 can promote the activation of PI3K-Akt signaling to induce the expression of P-glycoprotein, which involves in the absorption, distribution and excretion of chemical compounds and is regarded as a primary factor to induce acquired resistance to oxaliplatin [151].

In human HCC cells, ectopic expression of STC2 compromises paclitaxel-induced apoptosis through upregulating P-glycoprotein and Bcl2. Amphiphilic cationic PHB-PDMAEMA polyester has been synthesized and applied as a delivery carrier for paclitaxel to overcome P-glycoprotein-mediated resistant effects [162]; cell culture analysis demonstrated that this treatment partially overcomes STC2-P-glycoprotien-mediated drug resistance, providing a new way to more efficiently treat human cancers.

Hyperactivation of epidermal growth factor receptor (EGFR) signaling is a critical contributor for the development and progression of lung cancer [163]; therefore, TKIs are widely applied to treat non-small cell lung cancer (NSCLC) with the hyperactivation of EGFR signaling; however, some tumours will finally develop acquired resistance to TKIs. cDNA microarray analysis demonstrated the upregulation of STC2 in resistant tumour cells comparing to their parental counterparts, and STC2 is upregulated in several other cell lines exposed to TKIs, indicating that STC2 upregulation is a common phenomenon in resistant tumour cells [43]. Importantly, elevated STC2 levels are correlated with poor prognosis in patients with lung cancer. Moreover, ectopic expression of STC2 drives acquired resistance, while knockdown of STC2 successfully re-sensitizes resistant cells to TKIs. Mechanistically, STC2 enhances the activation of c-Jun singling, the transcription of AXL and the activation of Erk pathway [95]. Consistently, knockdown or chemical inhibitors targeting either AXL or Erk can facilitate overcoming TKI resistance-mediated by STC2 overexpression.

### STC2-mediated resistance to radiotherapy

Radiotherapy or radiation therapy is an approach to treat tumours through exposing them to high dosages of radiation. Mechanistically, radiation causes DNA damage-induced cell death, and finally suppresses tumour growth. Radiation therapy includes external beam, internal beam and systemic radiation like radioactive iodine for thyroid cancer, and molecular radiotherapy for advanced prostate cancer and gastroenteropancreatic neuroendocrine tumour. External beam radiotherapy is widely applied to treat many different tumours including breast cancer, colorectal cancer, esophageal cancer, HNSCC, NSCLC, skin squamous cell carcinoma, prostate cancer, brain tumours; while internal beam therapy is normally used to treat HNSCC, breast cancer, cervical cancer, prostate cancer and tumours from the eye. Although they are sensitive to radiotherapy initially, tumour cells will finally develop acquired resistance to radiotherapy. Therefore, it is critical to identify new molecular mechanisms of how tumour cells develop acquired resistance. As a stress response factor, STC2  is upregulated upon irradiation treatment [164, 165]. In a study with human nasopharyngeal carcinomas, after intensity-modulated radiotherapy, the incidence rate of residual tumours is 54.8% for patients with STC2 positive tumours, while it’s only 17.4% for those with STC2 negative ones, suggesting STC2 levels are correlated with the response of tumours to radiation therapy [38]. Consistently, these findings are also revealed in patients with cervical cancer [46]. Another study utilized human CNE2 nasopharyngeal carcinoma cells to investigate the mechanism of how STC2 mediates the development of acquired resistance to radiation. STC2 knockout compromises colony formation induced by radiation regardless of oxygen levels because of increased apoptosis and reduced cell proliferation [79]. Further analysis indicates STC2 knockout not only promotes cell cycle arrest in G_2_-M phase, but also suppresses cell migration and invasion. Therefore, STC2 is an indispensable factor to control post-radiation proliferation, survival and migration; and STC2 is a promising target for developing a novel therapy to overcome radiation resistance in human cancers.

### Conclusions and future perspectives

STC2 is upregulated in most types of human cancers. Elevated STC2 levels are associated with tumour size, invasiveness, metastasis and poor prognosis in most tumours other than some breast cancers. In addition, overexpression of STC2 is controlled by diverse mechanisms that will fit STC2 into different signaling networks in order to facilitate cell survival. Further analyses are required to dissect these mechanisms in a context-dependent manner.

As a secreted glycoprotein hormone, serum STC2 levels can be easily monitored, thus it is promising to investigate whether serum STC2 levels can be utilized as a practical biomarker to assess prognosis or recurrence in patients with various types of cancers. STC2 regulates various aspects of tumour biology like cell proliferation, invasion and metastasis that are associated with tumour phenotypes and disease stages. Solid tumour cells commonly encounter stress conditions which selectively activate corresponding signaling pathways to reprogram metabolism, adjust tumour cell survival, proliferation, invasion and migration. STC2 is upregulated in many tumour cells under multiple stress conditions which activate ATF4, HIF-1 or both, strongly suggesting that STC2 participates in the evolutionarily conserved response under stress conditions.

STC2 is a glycosylated and secreted protein; hence, it is hypothesized to function in an autocrine or paracrine manner, but its potential receptor remains unclear [166]. The primary and most critical question is to identify STC2 receptor in order to better understand its signal transduction pathway and mechanisms of action. In addition, intracellular functions of STC2 prior to secretion also warrant further investigation.

In summary, STC2 is a promising biomarker to evaluate patients’ prognosis and a potential candidate for developing targeted therapies, particularly for patients with late-stage diseases that usually require combined therapies. Further investigation into its mechanism of action may facilitate its clinical utilization as a biomarker and a novel therapeutical target.

## Data Availability

Not applicable.

## Abbreviations

AhR: Aryl hydrocarbon receptor
ATF: Activating transcription factor
CRPC: Castration-resistant prostate cancer
CTC: Circulating tumour cells
DFS: Disease-free survival
ECM: Extracellular matrix
EGFR: Epidermal growth factor receptor
EMT: Epithelial-mesenchymal transition
Er: Estrogen receptor
ER stress: Endoplasmic reticulum stress
ErbB: Epidermal growth factor receptor
ESCC: Esophageal squamous cell carcinoma
FISH: Fluorescence in situ hybridization
GCN2: General control nonderepressible 2
HCC: Hepatocellular carcinoma
HDAC: Histone deacetylase
HIF: Hypoxia-inducible factor
HMGA2: High-mobility gene group A2
HNSCC: Head and neck squamous cell carcinoma
HRE: Hypoxia-response element
HRG: Histidine-rich glycoprotein
HRI: Heme-Regulated Inhibitor (= Eukaryotic Translation Initiation Factor 2 Alpha Kinase 1)
IHC: Immunohistochemical
IRE1: Inositol-requiring protein 1
lncRNA: Long non-coding RNA
miRNA: MicroRNAs
MMP: Matrix metalloproteinase
NSCLC: Non-small cell lung cancer
PERK: PKR-like ER kinase
PKR: Protein R kinase **(= **Eukaryotic Translation Initiation Factor 2 Alpha Kinase 2)
qRT-PCR: Quantitative Real-Time Polymerase Chain Reaction
RCC: Renal cell carcinoma
Sp1: Specificity protein 1
STC: Stanniocalcin
TKI: Tyrosine kinase inhibitor
UPR: Unfold protein response
UTR: Untranslated region
VEGF: Vascular endothelial growth factor
